# Hotspots of soil organic carbon storage revealed by laboratory hyperspectral imaging

**DOI:** 10.1038/s41598-018-31776-w

**Published:** 2018-09-17

**Authors:** Eleanor Hobley, Markus Steffens, Sara L. Bauke, Ingrid Kögel-Knabner

**Affiliations:** 1 Soil Science, Technical University of Munich, Weihenstephan, Germany; 2 Research Institute of Organic Agriculture FibL, Frick, Switzerland; 3 Institute of Crop Science and Resource Conservation, Soil Science and Soil Ecology, University of Bonn, Bonn, Germany; 4 Institute for Advanced Study, Technical University of Munich, Garching, Germany

## Abstract

Subsoil organic carbon (OC) is generally lower in content and more heterogeneous than topsoil OC, rendering it difficult to detect significant differences in subsoil OC storage. We tested the application of laboratory hyperspectral imaging with a variety of machine learning approaches to predict OC distribution in undisturbed soil cores. Using a bias-corrected random forest we were able to reproduce the OC distribution in the soil cores with very good to excellent model goodness-of-fit, enabling us to map the spatial distribution of OC in the soil cores at very high resolution (~53 × 53 µm). Despite a large increase in variance and reduction in OC content with increasing depth, the high resolution of the images enabled statistically powerful analysis in spatial distribution of OC in the soil cores. In contrast to the relatively homogeneous distribution of OC in the plough horizon, the subsoil was characterized by distinct regions of OC enrichment and depletion, including biopores which contained ~2–10 times higher SOC contents than the soil matrix in close proximity. Laboratory hyperspectral imaging enables powerful, fine-scale investigations of the vertical distribution of soil OC as well as hotspots of OC storage in undisturbed samples, overcoming limitations of traditional soil sampling campaigns.

## Introduction

Storing around 1500 Pg of organic carbon (OC) in the upper 1 m of soil^1^, soils comprise the largest stock of the terrestrial C pools. Understanding soil organic carbon (SOC) storage and dynamics is therefore crucial to our understanding of the system Earth and how humans interact with and influence our environment. Unfortunately for scientists aiming to assess the response of soils to environmental and management changes, the spatial variability of SOC storage is high, limiting our capacity to detect changes in SOC across various scales. Coefficients of variation of SOC storage exceed 50% across landscapes^2^ and within some soil types relative variance can exceed 200% globally^1^. Even at the microscale, SOC storage is highly heterogeneous, and microscale variability has been linked with soil formation^3^ and soil functionality^4^, so it should not be dismissed when investigating SOC dynamics.

Contributing to its complex spatial heterogeneity is the great variability of SOC with profile depth. In many soils, OC storage decreases with increasing soil depth, and subsoils - i.e. the soil located below the ‘topsoil’ (surface) horizon have been frequently excluded from investigations into SOC storage and dynamics. Nevertheless, with the realisation that large quantities of C are stored in subsoils^5,6^ and that subsoil C is dynamic^7^, interest is growing in investigating SOC dynamics not only in surface soils but further down the soil profile. In particular, the influence of agriculture and human management on subsoil OC storage and dynamics is a field of growing interest^8,9^.

Unfortunately, detecting significant changes in C content in subsoils is difficult due to the generally reduced OC contents in subsoils, which result in smaller magnitudes of change in OC storage in subsoils - for example due to land-use change or specific management practices^10^. Furthermore, in contrast to surface horizons of agricultural soils, it is rarer for subsoils to be mixed, e.g. during ploughing^11^. As such, subsoils are often characterised by pronounced heterogeneity^12–14^, with distinct regions of enhanced or depleted SOC storage^15^. Such hotspots are potentially important for SOC storage, but against the backdrop of generally low OC contents in deeper soil horizons, this greater variance of subsoil OC storage renders it more difficult to detect significant differences in SOC storage between sites of interest. Despite these difficulties, subsoils remain important to SOC storage^16^ and should not be excluded from SOC inventories, so that new approaches are required to assess SOC changes at depth.

Classical sampling methods for SOC investigations involve sampling discrete depth intervals or horizons and subsequent mixing of the samples. This homogenisation is important to overcome the microscale variability of soil^3^, enabling analysis of representative subsamples which reflect the SOC contents across field sites or landscapes. However, homogenization masks the presence of hotspots or coldspots in the profile, only enabling overall trends in the depth distribution to be investigated. As such, homogenization implies a loss of information content in the sample, in particular regarding spatial variability in SOC distribution within the sample.

Similarly, sampling discrete depth intervals or soil horizons reduces the vertical resolution of information content, as sample information is averaged per depth interval. Volumes of subsoil samples are frequently larger than surface samples, as depth intervals in subsoils are often larger than in surface horizons^10,17^. This larger volume results in an even greater reduction in sample information content after mixing, implying a further loss of statistical power. To overcome this low statistical power, a greater number of samples is required, which implies greater analysis cost and effort.

Various methods are employed to overcome this reduction in information content down the soil profile. Sampling can occur at the profile face, with fine sampling grids used to sample the profile at higher resolution than traditional methods^18^. However, fine-scale profile sampling is timely and labour-intensive, as well as highly destructive, thereby hindering its wide-spread adoption and application in soil investigations.

Sampling of soil cores is a more rapid, less labour-intensive approach to soil sampling, which is frequently applied in SOC investigations, although cores are traditionally cut into discrete depth increments - usually ~10–30 cm - and the obtained samples homogenized. More recently, spectroscopic investigations of soil cores have been presented^19,20^, facilitating a rapid, information dense investigation of the vertical distribution of soil properties at the cm scale.

Hyperspectral imaging of soil profiles in a laboratory setting provides the possibility of further increasing the resolution of accessible soil information, providing information on the vertical and horizontal distribution of C in the soil profile at the micro-scale^21^. With hyperspectral imaging, each pixel has an associated spectrum, so that the spectral variability of the sample can be assessed at the spatial resolution of the image. Given the strong association of infrared (IR) spectra with SOC content^22^, this spectral information can potentially be used to derive SOC spatial distribution in an undisturbed soil sample. Although laboratory hyperspectral imaging is promising for assessing the information content of soil at very high scale resolution, its application to date has been largely limited to near surface horizons^23,24^ and its use in deep subsoils is yet to be thoroughly tested.

A variety of modelling approaches are used to predict SOC from spectral information, with the ‘classical’ partial least squares (PLS) regression^25^ most frequently applied. The PLS algorithm fits a linear multivariate model to the data by projecting both the predictors (i.e. spectral response) and response variable (SOC) to a new dimension to identify latent covariance structures^26^. However, relationships between soil spectra and SOC contents are frequently non-linear^27^, which may present a difficulty for linear regression approaches.

The advent of non-linear data mining algorithms has introduced the potential for different non-linear modelling applications to soil spectroscopy^28^. Two such data mining algorithms are random forest (RF)^29^ and support vector machines (SVM)^30^. Random forests are stochastically driven tree-based models which recursively partition a response variable into groups of increasing purity based on the predictor variables. Support vector machines, on the other hand, construct very high dimensional hyperplanes to separate data in the feature space and produce model estimates. These different machine learning approaches may be more suited to predict the non-linear relationships between soil spectral response and SOC contents^31^. Alternatively, data transformations may also help to overcome issues associated with modelling SOC contents based on sample spectra and are frequently used to improve predictive performance of spectrally based models^32^.

In this study we investigate the application of laboratory hyperspectral imaging to predict SOC contents down the soil profile on cores sampled down to 1 m from an agricultural site near Bonn, western Germany. We hypothesize that, due to its high resolution (typical pixel resolution <100 × 100 µm depending on the instrument), hyperspectral imaging in the visual - near infrared (VNIR) domain is capable of capturing the fine-scale spatial variance in spectral properties down the profile, which combined with multivariate modelling is a suitable tool to predict profile SOC distribution at the fine-scale. Our aims were thus to (1) assess the suitability of hyperspectral imaging combined with several modelling (PLS, RF, SVM) and spectral transformation approaches (normalization, standard normal variate (SNV) transformation) for predicting SOC content down the soil profile, (2) to assess the SOC content and its variance down the profile at the micro-scale, (3) to test the models’ capacity to identify localised regions of higher or lower SOC storage (i.e. hotspots or coldspots).

## Results and Discussion

### Laboratory hyperspectral imaging of soil cores and selection of regions of interest for calibration

**Figure 1:**
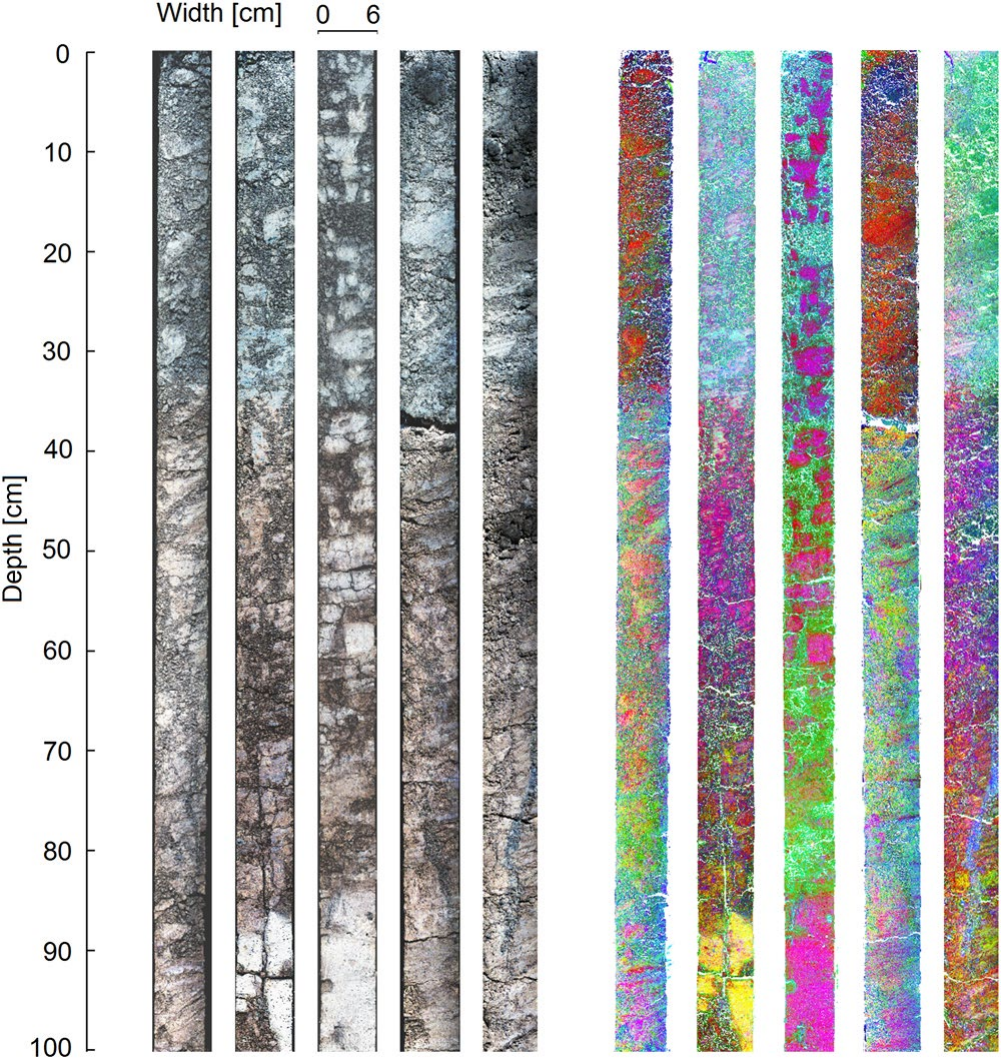
Hyperspectral images of the soil cores. Left: images normalized to reflectance target with RGB bands rendered using three bands in the visual range of the spectrum (red: 580 nm, green: 550 nm, blue 450 nm). Right: first three principal components calculated on individual cores displayed as RGB bands (red: principal component 1, green: principal component 2, blue: principal component 3).

The normalized hyperspectral images of the soil cores were dominated by regions of brightness and darkness throughout the cores (Fig. 1). Principal components analysis performed for each soil indicated fine-scale qualitative differences in spectral properties of the cores (Fig. 1). In particular, there were distinct differences in the spectral properties of the topsoil and subsoil in each analysed core. Additionally, spectrally distinct regions were identifiable at all depths, though in particular in the subsoil. These regions were, along with visually identified differences in soil core surface properties, used to select the regions of interest (ROI) for calibration. This enables variation in the calibration samples to be maximised without prior knowledge of the quantities and distribution of SOC in the soil core, helping to optimize the calibration.

### Spectral treatment effects on SOC prediction models

**Figure 2:**
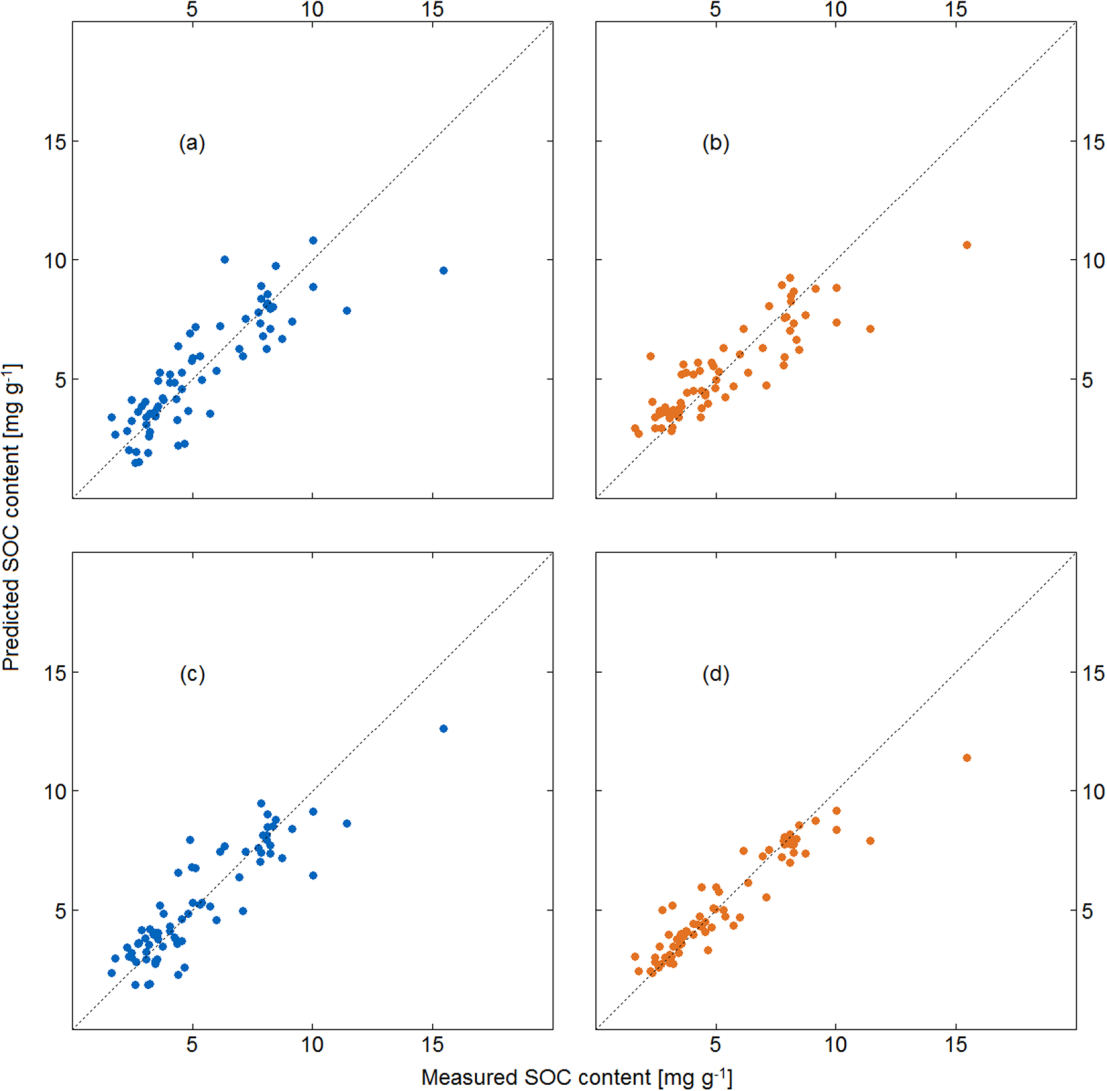
Predicted vs. measured SOC content of the calibration (ROI) dataset for (**a**) partial least squares predictions using the normalized reflectance spectra, (**b**) random forest predictions using the normalized reflectance spectra, (**c**) partial least squares predictions using the standard normal variate spectra, (**d**) random forest predictions using the standard normal variate spectra.

The cross-validated estimates of the PLS and RF models of ROI spectra to SOC contents indicated that z-scoring spectra to produce standard normal variate spectra substantially improved model performance (Fig. 2). The PLS and RF algorithms fit to the normalized reflectance (NR) spectra produced very similar results, with both models having low bias <0.001 mg g^−1^, root mean squared error (RMSE) = 1.3 mg g^−1^, mean absolute error (MAE) = 1.0 mg g^−1^, residual prediction deviation (RPD) = 2.0. The coefficient of variation was also very similar, with R² of 0.75 and 0.76 for PLS and RF respectively. Fitting the models to SNV spectra improved performance for both algorithms, with RF outperforming the PLS model after SNV transformation. The validation estimates of the PLS model fit to SNV spectra indicated very low bias <0.001 mg g^−1^, with RMSE = 1.0 mg g^−1^, MAE = 0.8 mg g^−1^, RPD = 2.6 and R² = 0.85. The validation of the RF model fit to the SNV spectra also had low bias <0.01 mg g^−1^, RMSE = 0.8 mg g^−1^, MAE = 0.5 mg g^−1^, RPD 3.3 and R² = 0.91. This improvement to both the PLS and RF models concurs with previous findings^33^ reporting improved predictive accuracy of hyperspectral imaging spectroscopy after spectral pre-processing.

The improvement in model performance may be the result of the sensitivity of hyperspectral imaging to variations in core surface properties. NIR spectroscopy is sensitive to light scattering, which is affected by differences in surface properties of the sample^34^, e.g. resulting from differences in grain size in a sample or due to minor difference in distance from lens at different locations in a core. We aimed to minimize this effect by sampling both flatter and rougher surfaces as well as sampling different locations and heights from within the cores, thereby incorporating this variance in sample surface properties into the calibration models.

However, ROI should be as small as possible, so that their variance is minimized and they reflect ‘pure’ regions within the soil cores, which contain homogenous and representative soil material. Given these constraints, it was not possible to fully capture variance in surface properties within the calibration samples whilst simultaneously minimizing the size of the ROI. Standardising spectra to the mean and dividing by the standard deviation (SNV transformation) helps to overcome these differences in light intensity arising from uneven surface properties in the soil cores, as each data point in a spectrum is not absolute but relative to the other data points within the spectrum, which accounts for the improvement in model performance after z-scoring. The greater improvement after z-scoring the spectra in the RF model indicates that this non-linear modelling approach is more sensitive to such spectral scattering than the linear regression algorithm underlying the PLS model.

Due to the clear improvement in predictive performance of the PLS and RF models built using the SNV spectra^32^, the further results and discussion present only the models built using SNV spectra for these algorithms.

### Model predictive performance

#### Partial least squares algorithm

Applying the PLS algorithm to predict SOC in the whole soil cores resulted in a large number of cells with negative OC contents (Table 1), with the proportion of negative cells per core between 20% and 27%. Similarly, the PLS algorithm predicted values outside the upper limit of the analysed data, with between 8 and 25% of predictions lying above the highest C content of any analysed sample. As a result, between 29 and 49% of the data predicted by the PLS algorithm in individual cores were implausible, with overall 48% of data points unreliable. Further, this resulted in high variance within the predictions which greatly exceed the variance of the SOC contents of the bulk cores and ROI.

**Table 1:** Statistical properties of OC in cores analysed and predicted with different model algorithms.

Data	Mean [mg g^−1^]	Median [mg g^−1^]	Maximum [mg g^−1^]	Minimum [mg g^−1^]	Standard deviation [mg g^−1^]	Proportion < 0	Proportion > upper limit
Regions of interest	5.3	4.6	15.5	1.6	2.6	0	0
PLS	6.4	6.1	562	−187	9.6	0.23	0.15
PLS limit constrained	7.1	6.1	15.5	1.6	5.2	0	0
SVM	5.7	5.8	9.6	2.6	0.4	0	0
RF	5.8	5.7	14.7	2.0	2.0	0	0
RF bias corrected	5.3	4.5	16.1	0.5	3.0	0	0

This tendency to predict outside the range of calibration is a well-documented drawback of the PLS algorithm^35,36^, which limits the application of such models for predictive purposes. This arises from the linear extrapolation of the model to spectra beyond its calibration limits. Given the non-linearity in spectral response to linear changes in SOC contents^27,37^, the poor distribution of the PLS predictions suggests that for hyperspectral data similar to those presented here, where due to the data size the number of training points is a small fraction of the whole data set, the PLS algorithm is less suited for predictive purposes.

In order to overcome this limitation, we constrained the PLS predictions by replacing the values less than 0 with the minimum SOC content analysed in the calibration, and the values outside of the upper range of the calibration with the maximum SOC content analysed in the calibration. The distribution of the constrained PLS model predictions better reflected the distribution of the analysed SOC contents in the ROIs, and was also characterized by lower variance (Table 1). However, the mean and median values for the data were too high, and were reflected in an increase in model bias and poor goodness-of-fit statistics (Table 2). The large differences between the goodness-of-fit for the calibration validations and the evaluation based on the whole soil cores indicates that these models were overfit. As such, we deemed the PLS model unsuitable for predicting SOC contents from the hyperspectral data in these cores.

**Table 2:** Model goodness-of-fit for SOC predicted using SNV spectra with different model algorithms and evaluated against SOC analysed in full soil cores. ME: mean error, RMSE: root mean squared error of prediction, MAE: mean absolute error of prediction, RPD: residual prediction deviation, R²: model explained variance.

Model	ME	RMSE	MAE	RPD	R²	ME	RMSE	MAE	RPD	R²
	Modelled mean SOC					Modelled mean SOC ± 1 sd				
PLS	−1.7	2.1	1.8	0.99	−0.02	0	0	0	∞	1
PLS limit constrained	−2.6	2.9	2.7	0.71	−1.00	0	0	0	∞	1
SVM	−1.3	2.2	2.1	0.91	−0.21	−0.1	2.0	1.8	1.04	0.07
RF	−1.2	1.8	1.6	1.10	0.18	−0.3	0.7	0.4	2.75	0.87
RF bias corrected	−0.2	0.9	0.7	2.31	0.81	−0.0	<0.1	<0.1	25.5	>0.99
	**Modelled median SOC**					**Modelled median SOC** ± **1 sd**				
PLS	−1.5	1.9	1.6	1.06	0.12	0	0	0	∞	1
PLS limit constrained	−1.5	1.9	1.6	1.06	0.12	0	0	0	∞	1
SVM	−1.3	2.3	2.1	0.90	−0.25	−1.0	2.0	2.0	1.02	0.04
RF	−1.0	1.7	1.5	1.19	0.29	0.4	0.7	0.4	3.04	0.89
RF bias corrected	−0.0	0.8	0.6	2.42	0.83	0.0	<0.1	<0.1	20.6	>0.99

#### Support vector machine algorithm

In contrast with the high variance in predictions obtained using the PLS algorithm, the distribution of the SVM predictions was characterized by low variance, with the upper and lower values substantially within the range of the SOC analysed in the ROI (Table 1). This suggests that the algorithm was unable to capture and reproduce local variance in the core, and that the model is unsuited for identifying hotspots and coldspots in the cores. This is potentially a result of a poor optimization of the models, so that generalization to larger data sets becomes difficult. Nevertheless, optimization was tested and performed using both bootstrapping and cross-validation, which are generally proposed to be the best choice for model optimization purposes^38^. The large differences between the goodness-of-fit for the calibration validations (Section *Spectral treatment effects on SOC prediction models*) and the evaluation based on the whole soil cores (Table 2) indicates that these models were overfit. As such, the poor generalization of the models to the spectra from the entire cores suggests that these models are poorly suited for predictive purposes. This result is confirmed by the poor goodness-of-fit statistics, with large RMSE and RPD below 1 (Table 2). This concurs with previous findings as to the poor generalizability of SVM for predicting soil properties^28^ and suggests that these models are poorly suited for predicting SOC from hyperspectral imaging of these cores.

#### Random forest algorithm

The RF algorithm predictions were most similar to the distribution of SOC within the soil cores in terms of mean, median, extreme values and standard deviation (Table 1), indicating that the predictive properties of the algorithm reflect the general distribution of SOC within the cores. However, the RMSE was relatively high compared with the mean SOC content of the cores, resulting in relatively poor goodness-of-fit properties, though still better than the PLS and SVM predictions (Table 2).

After examining the residuals of the model, a relationship between model residuals and SOC content was apparent, with higher SOC contents underestimated, and lower SOC contents tending to be overestimated. This is a known phenomenon in random forest models^39^, which arises from the algorithm grouping data into terminal nodes of greatest purity. Hereby, the regression to the mean of each terminal node implies an under-estimation of higher responses (in this case SOC) and an over-estimation of lower responses.

In order to overcome this bias, a bias correction was applied. As we could not use the SOC content to adjust the bias, we tested whether bias could be corrected based upon location (vertical and horizontal) in the cores. There was no relationship between horizontal location and bias. However, there was a non-linear relationship between residual bias and vertical location within a core, with topsoil SOC contents underestimated by 1.5 mg g^−1^ on average, and subsoil contents overestimated by 1.5 mg g^−1^ on average. As such, we bias corrected the predicted SOC contents (in mg g^−1^) arithmetically based on prediction location, i.e. topsoil vs. subsoil, with topsoil and subsoil visually identified using the first three principal components of the images:

$$SO{C}_{Topsoil,biascorrected}=SO{C}_{Topsoil}+1.5\,mg\,{g}^{-1}$$

$$SO{C}_{Subsoil,biascorrected}=SO{C}_{Subsoil}-1.5\,mg\,{g}^{-1}$$

**Figure 3:**
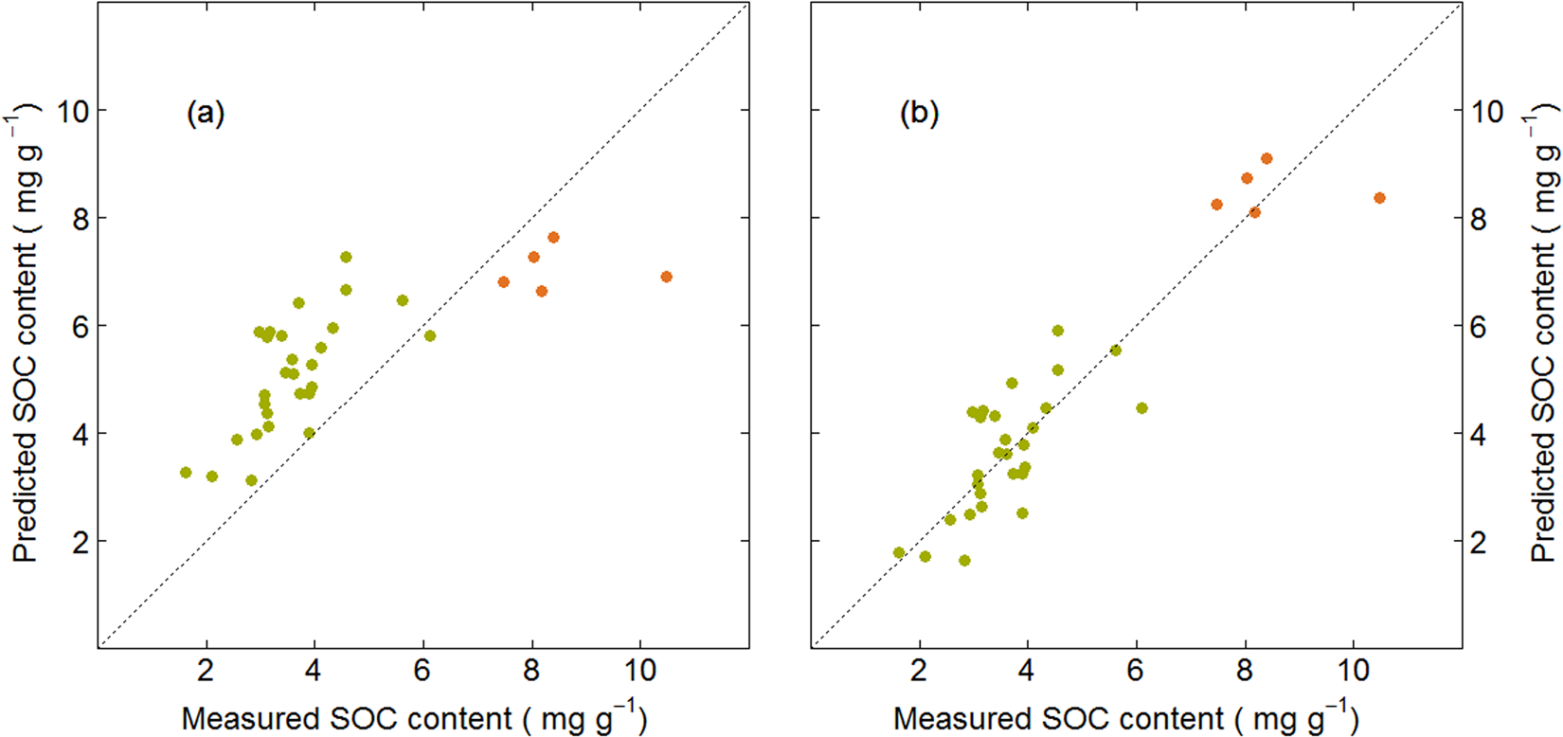
Predicted vs. measured SOC contents of the evaluation data set, i.e. based on SOC contents in the whole core, modelled via random forest (**a**) before and (**b**) after bias correction. Topsoil samples are indicated by orange dots, subsoil samples by green dots.

This bias correction improved the goodness-of-fit properties significantly, with bias essentially eliminated, and RPD > 3.0, indicating excellent predictive capacities^40^. Importantly, the RMSE and MAE for the calculations using both the mean and median SOC contents were ≤0.9 mg g^−1^, the limit of determination^41^ of C using the dry combustion method. The mean and median SOC contents as well as the standard deviation of the bias corrected RF predictions closely reflected those of the soil cores (Fig. 3), although the upper and lower range of the data were slightly outside the upper and lower range of calibration. For the maximum, the deviation was smaller than the RMSE of the prediction, so that the difference was not-significant. For the minimum, the deviation was marginally larger than the RMSE of prediction, but still not implausible for subsoil OC contents. Due to the overall good performance and closest fit to SOC distribution, the bias corrected RF was deemed most suitable for predicting SOC contents in the soil cores.

### Depth distribution of SOC in the soil cores

**Figure 4:**
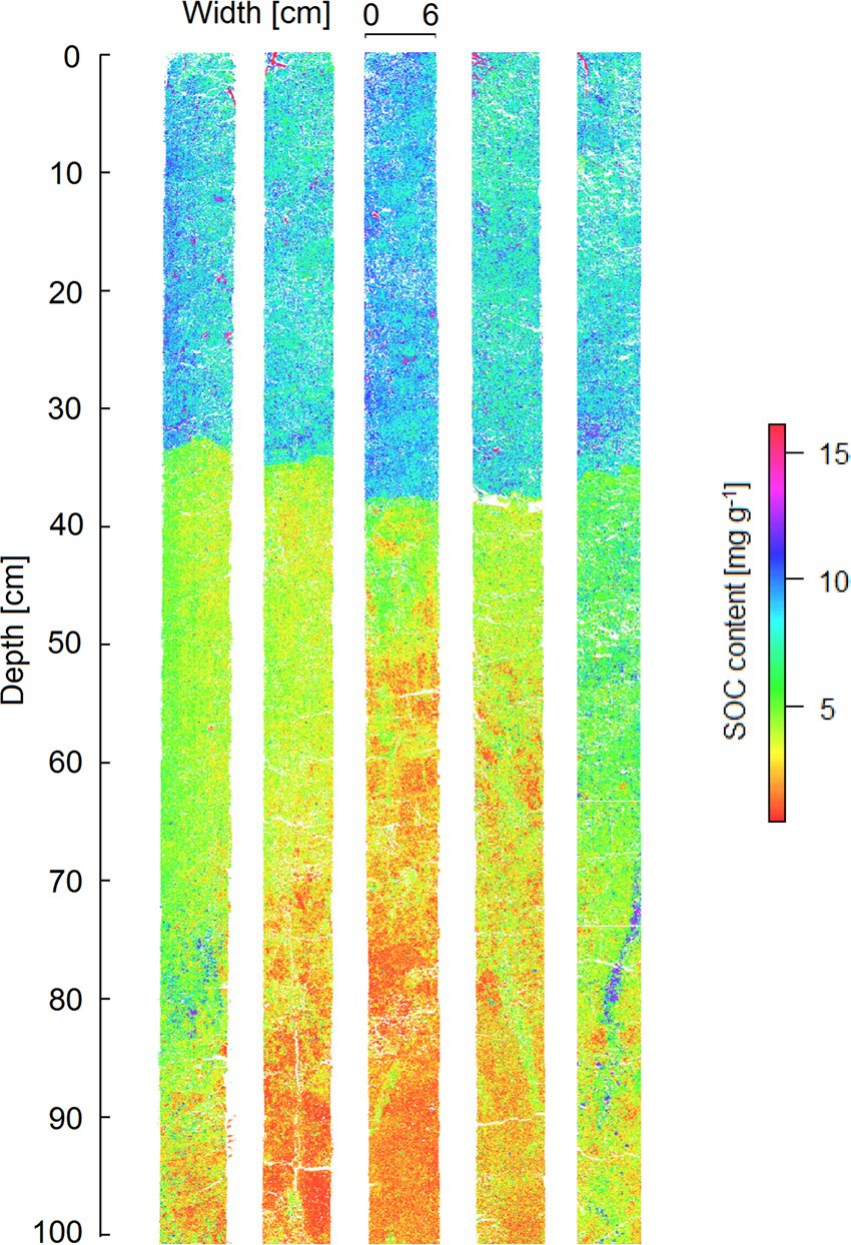
SOC distribution predicted using bias corrected random forests from hyperspectral images of five soil cores.

**Figure 5:**
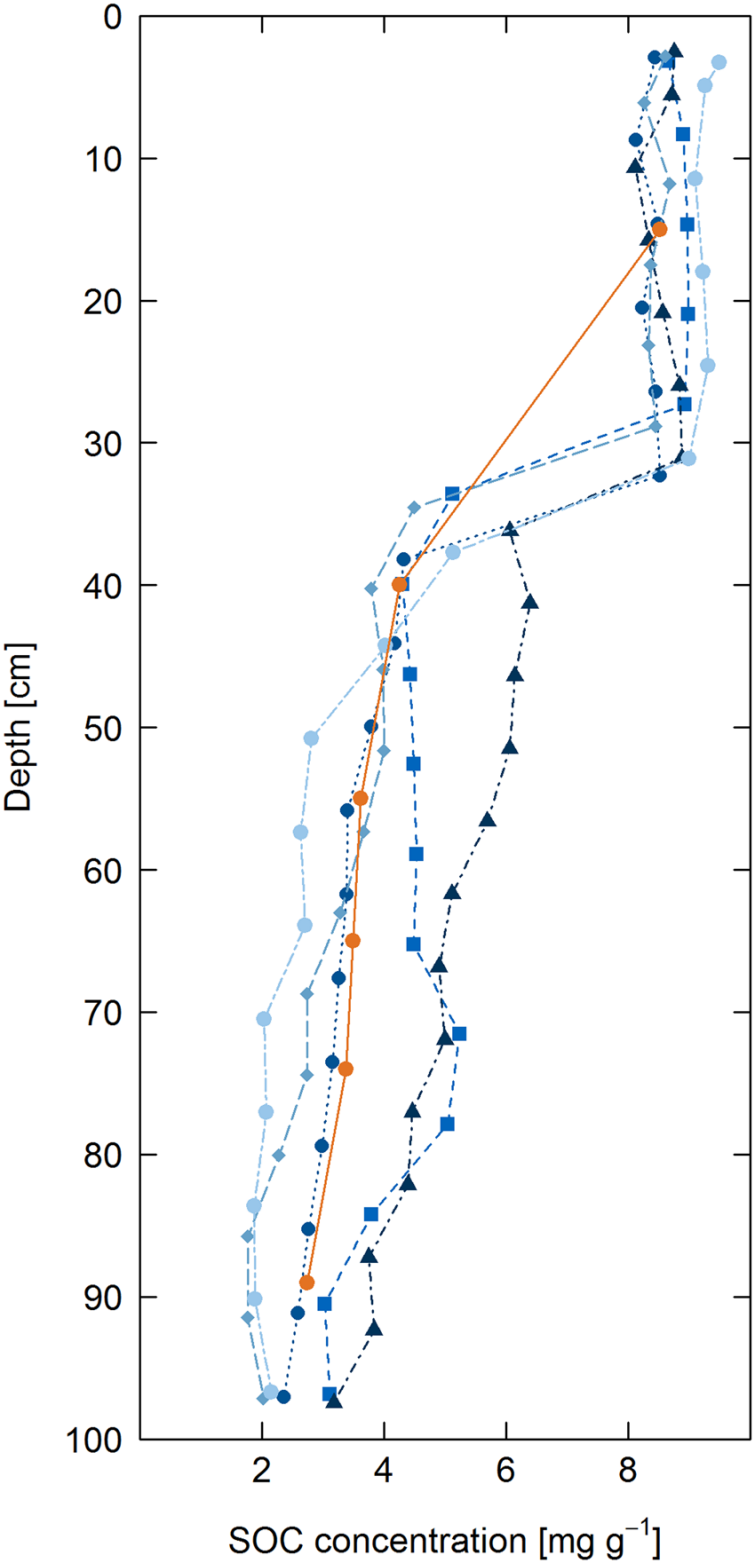
Vertical distribution of mean SOC contents measured in bulk core samples (orange) and SOC predicted using bias corrected random forests from hyperspectral images of individual soil cores (blue and black).

**Figure 6:**
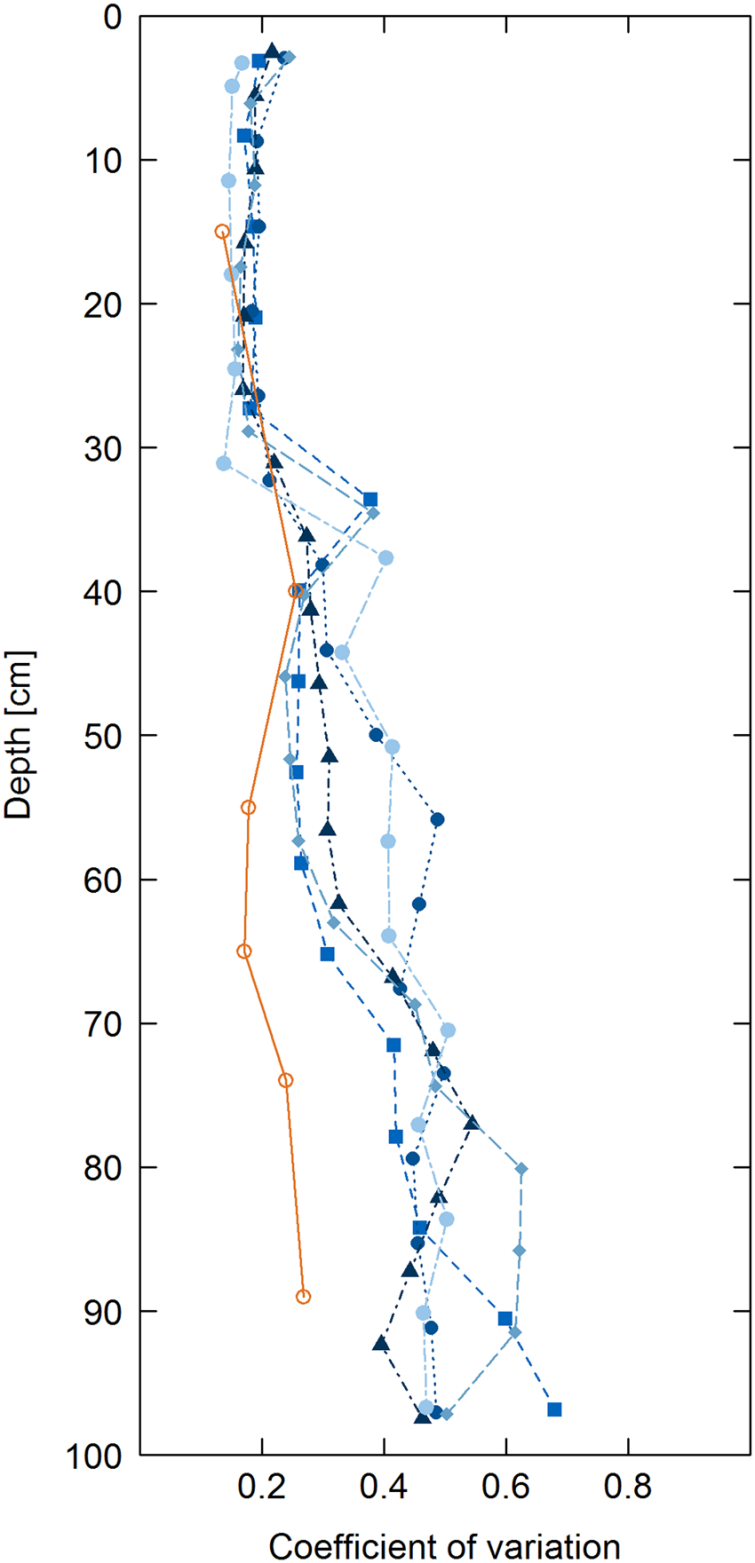
Vertical distribution of coefficient of variation of SOC across bulk cores (orange) and within cores calculated from SOC predicted using bias corrected random forests from hyperspectral images.

The bias-adjusted RF predictions of SOC in the soil cores revealed the highest mean SOC contents in the plough horizon and reduced SOC contents at depth (Fig. 4), which is consistent with general trends in SOC depth distribution for soils across the globe^5,42^. The mean SOC content in the plough horizon was relatively homogenous throughout the horizon at between 8 and 9 mg g^−1^, with a coefficient of variation of ~0.2 in predicted SOC in the soil cores (Figs 5 and 6). These predictions mirrored the mean SOC content analysed in the bulk soils, at 9 ± 1 mg g^−1^ across all cores, indicating good model performance.

These measured and predicted SOC contents in the plough horizon are lower than the mean topsoil OC content [29 mg g^−1^] of European soils^43^, but very similar to other topsoil OC contents [9–10 mg g^−1^] in the region^44^. Such low SOC contents in the surface horizon are consistent with the agricultural management of these soils, which tend to store significantly less OC than other land-use types^45^, with surface soils having the greatest discrepancies to soils under pasture and forest use^46,47^. Nevertheless, the SOC contents are lower than the mean SOC content [19 mg g^−1^] of cropland soils in other German regions^48^. This suggests that the site is depleted in SOC due to intensive agriculture and land-use^44,49^. Under prudent and on-going management, there may therefore be potential to sequester OC in the plough horizon.

Identification of depth distribution of SOC is important for assessment of global SOC storage^5^. In the subsoil, mean SOC contents were significantly lower than in the plough horizon, with a gradual decline in SOC content with increasing depth (Fig. 5). Using a traditional analytical approach, the vertical distribution of SOC contents in the bulk samples approximately followed an exponential fit^46,50^. However, the greater resolution of the hyperspectral predictions indicates that the depth distribution is not exponential. Rather, below the relatively homogeneous SOC distribution in the plough horizon, the reduction in SOC content declined approximately linearly with increasing depth. This highlights the power of hyperspectral imaging and modelling to investigate the vertical distribution of SOC. In a traditional survey, the SOC depth distribution would not have been accurately captured due to the smaller number of samples and unequal sampling depths, whereas the hyperspectral imaging spectroscopy is capable of reproducing the vertical distribution of SOC at high-resolution.

Using this hyperspectral imaging and data-mining technique, we can thus compare SOC contents at much finer scales. Although the calculations presented here used an equal area (~6 × 6 cm) basis to compare the mean and variance in SOC content down the profile, we imaged the SOC distribution at the micron-scale (53 × 53 µm pixel size), and so can reproduce SOC distribution at the micron-scale.

This can then be used to link profile SOC contents and distribution with the presence of hotspots or coldspots within the soil profile, and test whether they affect the mean SOC storage at the site. Importantly, due to the very high number of predictions per image (~23000000 per core, i.e. ~1200 pixels across a 6 cm soil core, ~19500 pixels down a 1 m core), the statistical power is much greater than in traditional analyses, helping to overcome the limitations of traditional subsoil analyses.

Subsoil OC distribution was more heterogeneous than in the plough horizon, although between the cores only a slight increase in coefficient of variation occurred with depth (Fig. 6). However, within each core SOC distribution was much more heterogeneous and increased with depth, with coefficients of variation increasing from ca. 0.2 in the plough horizon to a maximum of nearly 0.7 in the deep subsoil. This reflected the presence of localised hotspots and coldspots of SOC storage in close proximity (<1 cm distance) in the soil profile (Fig. 4).

**Figure 7:**
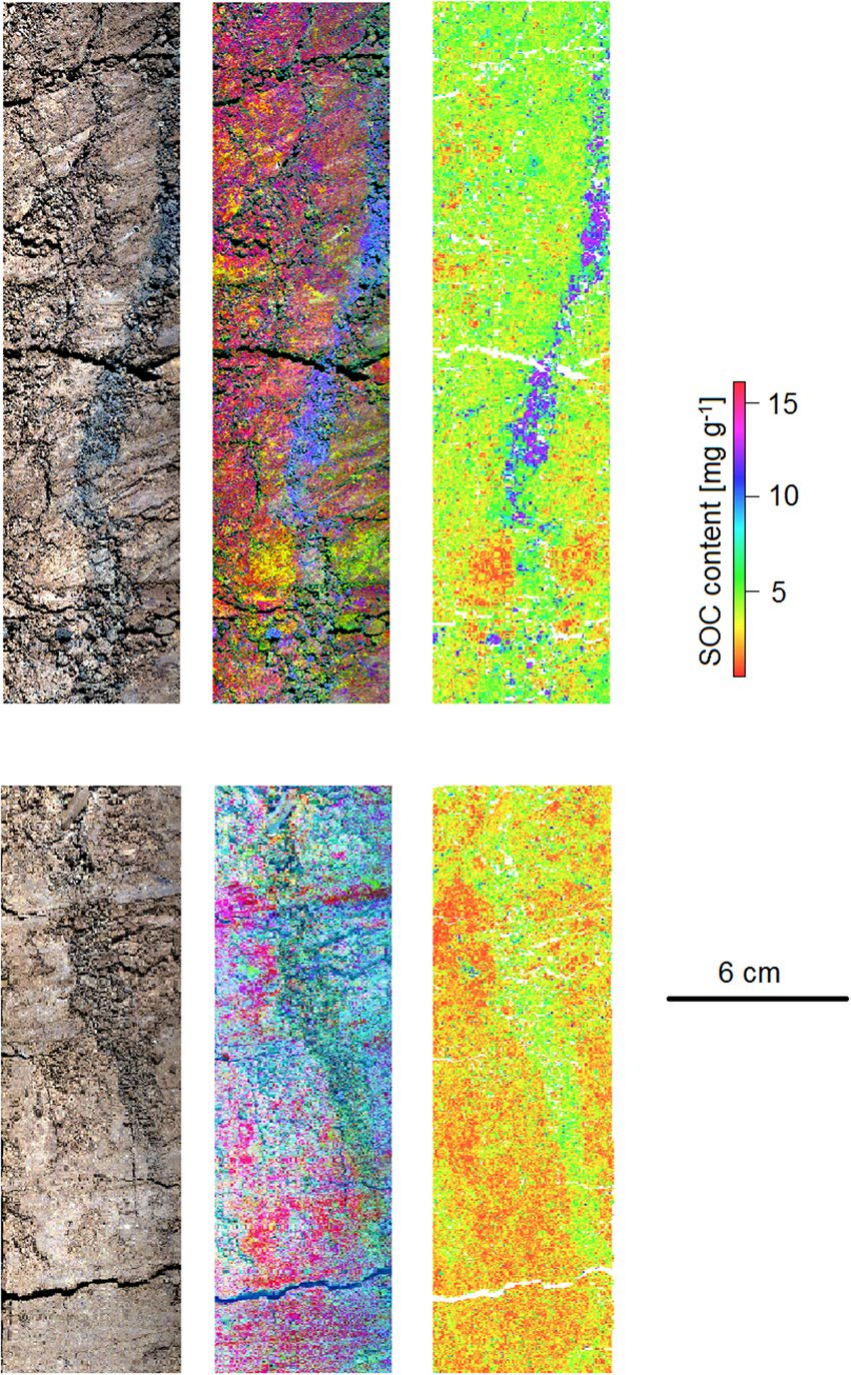
Subsoil biopores in different cores at a depth of ca. 69–83 cm (top) and 53–64 cm (bottom). Left: hyperspectral image with RGB bands in red, green and blue regions of visual spectrum. (red: 580 nm, green: 550 nm, blue 450 nm). Middle: first three principal components of hyperspectral image (red: principal component 1, green: principal component 2, blue: principal component 3). Right: predicted SOC content using a bias corrected random forest algorithm.

Small local hotspots of SOC were identified in the plough horizon, which were visually identified as plant roots and crop residues. In the subsoil, we were able to identify and quantify subsoil SOC hotspots which no longer consisted of visible plant material, namely biopores. These hotspots contained significantly larger quantities of SOC than directly adjacent soil compartments and confirm the importance of biopores for subsoil SOC storage. The highest measured SOC content [16 mg g^−1^] of any region of interest analysed - including all plough horizons - was associated with a subsoil biopore at a depth of ~69–83 cm (Fig. 7). At a distance of <1 cm from this biopore, the adjacent subsoil had SOC contents of 2–4 mg g^−1^, indicating enrichment of SOC in the biopore by a factor of 4 to nearly 10. Another biopore located in a different core at a depth of ~53–64 cm had lower SOC contents [6 mg g^−1^] but was still enriched by a factor of >2 compared with directly adjacent bulk soil [2 mg g^−1^]. This enrichment of C in biopores corresponds well with C enrichment in root biopores found in a similar agricultural soil in the region^51^, but is slightly lower than the ten-fold enrichment in SOC content in biopores in a sandy forest soil^14^. Based on the blurred delimitation between biopores and the surrounding soil matrix, we believe these biopores are old root channels.

This enrichment of SOC in subsoil biopores is consistent with spatially distinct regions of SOC stabilization in the soil profile^15,52^. In forests soil, SOC hotspots have been associated with root biomass and necromass, and silt content, although only in subsoil compartments^14^. Moreover, the rhizosphere plays an important role in subsoil OC storage and stabilization, in particular in soils derived from finer-textured, nutrient rich parent materials^53^. Our results suggest that this holds true for agriculturally managed, cropped soils, too, with root channels leading to the formation of biopore hotspots of SOC enrichment. Specifically, although localised hotspots of OC storage can form throughout the soil profile in cultivated soils, tillage disrupts, mixes and destroys these hotspots in the plough horizon, whereas they persist in non-mixed subsoil horizons.

Interestingly, the core with the very highly OC enriched subsoil biopore had the highest mean SOC content in the upper subsoil (30–60 cm, Fig. 5, black line). Given the 2-dimensional, surficial nature of the hyperspectral imaging technique, it is likely that the biopore extended into the soil core, not captured in the images. This implies that subsoil OC hotspots enhance mean SOC content despite their localised spatial extent, so that management approaches aimed at promoting subsoil biopore formation may lead to an overall increase in OC storage. This is potentially achievable via planting of deep rooting crops such as lucerne^52^, measures to promote anecic earthworm communities such as tillage reduction^54^ or compost application^55^, or burial of organic material in the deep profile^56,57^.

Importantly, traditional analysis of bulk cores, which are cut into discrete sampling depths and homogenized, prevents the identification of such hotspots and the investigation of their importance for SOC storage. The hyperspectral imaging technique presented here opens up the possibility of analysing SOC distribution at very high spatial resolution. This information can be used to identify and assess the contribution of hotspots located within the 2D imaged surface to total SOC storage, as well as to investigate the vertical distribution of SOC. Due to the high-resolution and therefore large number of predictions per image, the method provides statistically powerful results which can overcome traditional limitations of heterogeneity and low SOC contents in subsoils, enabling accurate assessment of SOC storage and dynamics at depth. Further, combination with other techniques (e.g. NanoSIMS) and potentially other nutrient analyses (e.g. Fe, N, P) can provide high resolution assessment of spatial covariance of SOC with other soil elements at the nanoscale, helping to elucidate fine-scale drivers of SOC storage. Hyperspectral imaging combined with data-mining is a powerful tool for investigating soil organic carbon spatial distribution.

## Methods

### Site description

The field experiment is located at the ‘Campus Klein-Altendorf’ research station of the University of Bonn, Germany, at 176 m a.s.l. (50°37′N, 6°59′E). The soil at the site is a haplic Luvisol^58^ developed from Loess deposits, the calcareous parent material can still be found below ca. 130 cm depth. The texture of the soil was a silty clay loam at the surface, grading to a silty clay in the deeper profile. The sand content showed no depth gradient, and was 5 ± 1% throughout the profile. The silt content was 62 ± 4% in the Ap horizon and gradually decreased to 53 ± 2% at a depth of 78–100 cm, whereas clay content was 31 ± 5% at the surface, gradually increasing to 40 ± 2% at a depth of 78–100 cm (mean values of 5 profiles). The soil was neutral to slightly alkaline, with a pH (1:5 in H_2_O) of 7.6 ± 0.1 in the Ap horizon (6.7 ± 0.1 in CaCl_2_), increasing to 8.2 ± 0.1 at a depth of 78–100 cm (7.2 ± 0.2 in CaCl_2_). Soil salinity was low, with electrical conductivity (1:5 in H_2_O) of 56 ± 13 µS cm^−1^ at the soil surface and 39 ± 10 µS cm^−1^ at a depth of 78–100 cm. The site experiences temperate climatic conditions with a mean annual temperature of 9.4 °C and mean annual precipitation of 603 mm (observation period 1956–2014, climate station University of Bonn, https://www.cka.uni-bonn.de/standort/Klima).

### Sampling and sample preparation

Five soil cores were sampled to a depth of 1 m using a stainless steel hydraulic corer (Ø 6 cm). To prepare the cores for hyperspectral image analysis, the cores were placed on a half-pipe and carefully sliced vertically in half from the bottom to the top with a knife. One half of the core was reserved for hyperspectral imaging, whereas the other half of the core was used to determine carbon contents of the bulk samples. Care was taken to prevent contamination and smearing during slicing, as well as to minimise differences in height of the sliced core surface. Where necessary, the sliced cores were gently levelled with a fine brush and/or microspatula. One half of each core was air dried at room temperature. During slicing, the other half of each core was carefully divided into 6 or 7 depths: 0–30 cm, 30–45 cm, 45–50 cm, (or 30–50 cm), 50–60 cm, 60–70 cm, 70–78 cm, 78–100 cm. These depths correspond to the sampling depths of the German Agricultural Inventory (www.thuenen.de/en/ak/projects/agricultural-soil-inventory-bze-lw/), modified to accommodate the presence of subsurface horizons/textural changes identified during field classification of the soils. These samples are hence-forth referred to as ‘bulk samples’. Three cores were cut into 7 depth increments, with two cores separated into 6 depth increments, yielding a total of N = 33 bulk samples. The bulk samples were air-dried at room temperature prior to sieving at 2 mm and removal of visible roots.

### Hyperspectral imaging and image processing

The dried soil cores were scanned with a HySpex VNIR-1800 hyperspectral camera (Norsk elektro optikk, Norway) after automatic dark background correction. The camera was fitted with a 30 cm lens with 1800 detectors in-line with a field of view of ~9 cm (final spatial resolution of image ~53 × 53 µm per pixel). For each pixel, 186 bands in the region 400–990 nm were obtained at a spectral resolution of 3.17 nm. The cores were mounted on a pushbroom translation stage which was moved underneath the camera and two 150 W illumination lamps to obtain the image. Prior to sample image acquisition, a reference scan of a calibration target with defined reflectance (~50%) was obtained.

To account for potential differences in illumination at different horizontal locations in the core, the spectral intensity (I) of the raw images of the soil cores were normalised to the defined reflectance (R) of the calibration target for each wavelength (λ) and pixel (x):

$${R}_{Sample,\lambda ,x}=\frac{{I}_{Sample,\lambda ,x}}{{I}_{Target,\lambda ,x}}\cdot {R}_{Target,\lambda ,x,defined}$$

The individual images were masked^23^ using spectral response and ratios of bands in the upper (~980 nm) and lower (~420 nm) regions of the spectra to identify and mask the translation stage background, the sample holder, shadows, cracks and macropores. Masking was done by mapping the spectral intensity of the VNIR band at ~980 nm and the ratios of the spectral intensity of bands at 980 nm and 420 nm and comparing these with the original images. From this, thresholds in the spectral response and ratios were identified and used to mask non-soil components of the images. Both the normalised reflectance (‘NR’) and standardised normal variate (‘SNV’, i.e. spectra transformed via z-scoring) spectra were used for modelling purposes.

### Soil carbon analysis

Approximately 3 g of each dried bulk sample was finely ground using a ball-mill prior to duplicate total C analysis by dry combustion (Hekatech, Germany). Inorganic C (IC) was determined in bulk samples via calcimetry upon reaction with 4 M HCl^59^. Inorganic C content was low (mean 0.01%, maximum 0.05%), with 67% of samples containing no detectable IC. Soil organic C was calculated as the difference between total C and IC.

After hyperspectral imaging of the half-cores, 71 regions-of-interest (ROI) were sampled from the 5 cores. Regions-of-interest were selected based on visual inspection of the soil cores as well as by applying a principal component transformation to the hyperspectral images and inspecting the first 5 components to visually identify regions of contrasting variance in the cores. Samples were obtained down the entire length of the cores, with the shallowest ROI at a depth of 3 cm and the deepest ROI at 99 cm (mean ROI depth 53 cm). 25% of ROI were sampled from the Ap horizons, with the remaining 75% sampled from the subsoil. Care was taken that samples covered a wide range of depths and horizontal locations in the cores, as well as being from visually distinct surficial properties (e.g. flat regions or regions of identifiable aggregation). Sampling was undertaken using a microspatula to gently scratch a small area from the core surface or using tweezers to remove aggregates. Between 50 and 120 mg of sample was taken from ROI covering areas between 0.1 and 4.8 cm² and ground using a microspatula prior to duplicate analysis of total C content by dry combustion. Due to the small ROI sample size limiting IC analysis, SOC was calculated as the difference between total C and the IC determined in the corresponding bulk soil of the core and depth the sample was drawn from. Simultaneous with sampling, the regions of interest were marked in the hyperspectral image (ENVI software) for subsequent extraction of spectral properties.

### Modelling soil organic carbon content

#### Model optimization, fit and prediction of SOC in the soil cores

Three modelling algorithms were tested for predictive modelling of soil organic C distribution using hyperspectral imaging, namely PLS regressions, RF and SVM, all implemented within the R software^60^. Separate PLS and RF models were built using the spectral responses (NR or SNV) as predictor variables and SOC as the target regression variable. The SVM algorithm requires standardized predictors, so the SNV spectra were scaled between 0 and 1 prior to model fitting. Additionally, model averaging was tested as a potential means to improve predictive performance compared with individual model algorithm approaches.

Due to the large number of spectra in each ROI (mean 57910 pixels per region of interest, range: 2534–170451), spectra for model fitting and validation were extracted as (1) the mean of all spectra in the ROI, (2) mean spectra from 5 separate random subsets representing 1% of the total number of pixels in each ROI, (3) the median of all spectra in the ROI and (4) median spectra from 5 separate random subsets representing 1% of the total number of pixels in each ROI. Initial model development and testing indicated that, regardless of the fitting algorithm, mean spectra were better suited for predictive purposes than median spectra, so results here are limited to models built using mean spectra.

Models were trained using the SOC content of the ROI as the response variable and the spectral response at each wavelength as potential predictor variables. Given the variability within the ROI, we trained the model using a subset of 3 of the random spectra from each ROI as potential predictors.

The PLS models were optimised via 5-fold cross-validation to select the optimal number of components for predictive modelling by minimising the cross-validated mean-squared error of prediction. In the final models, 10 components were selected for use.

The RF models were built with 200 trees using 33% of the predictor data for model training and 67% used for out-of-bag evaluation purposes. The number of predictor variables was the square root of the total number of predictor variables (i.e. 14) with the minimum number of cases in a node for splitting set to 2 and the minimum number of cases in a terminal node set to 1.

The SVM models were optimized simultaneously for γ, C and ε parameters, across a range of potential parameter values based on both 10-fold cross-validation and bootstrapping. γ, C and ε define the influence range of the points in the SVM hyperplanes, the penalisation for misclassification of points against model simplicity, and the error tolerance in the model, which influences the number of vectors in the model, respectively. The SVM model parameters selected from optimization were γ = 0.01, C = 8 and ε = 0.1 with 114 support vectors.

Model validation for optimization purposes was performed (1) by bootstrapping the models using one third of the data for model training and two thirds of the data for model evaluation, (2) by predicting the mean spectra using the models and (3) by predicting the remaining two independently sampled random subset spectra. Given the only conditional independence of the spectra, which are independent of each other but not of the associated SOC content of the ROI, inflation of the goodness of model fit may occur.

After optimization, the PLS, RF and SVM models were applied to the hyperspectral images to predict C contents down the individual cores. To compare the distribution of predicted SOC contents down the profile, the mean, standard deviation, coefficient of variation and skew of SOC contents were calculated for equal areas (defined by equal number of vertical and horizontal pixels pro area) down each of the soil cores.

### Model evaluation

To overcome the potential issue of inflation of goodness-of-fit statistics, final model evaluation was based on extracting the predicted SOC contents down the whole soil cores corresponding with the sampling depths of the bulk samples and calculating goodness-of-fit using these samples. As the ROI constituted only 3.5% of the entire area of the cores, the influence of ROI training spectra on these samples is negligible, so that they are essentially independent and therefore suited for model evaluation purposes.

Model evaluation and assessment of goodness-of-fit was based on (a) distribution of predicted C contents compared with measured C, (b) model bias (mean error, ME), (c) root mean squared error of prediction (RMSE), (d) mean absolute error of prediction (MAE), (e) residual prediction deviation (RPD), (f) model explained variance (R²). Model evaluation statistics were calculated for the image regions corresponding with the bulk samples using (1) the mean predicted C contents, (2) the median predicted C contents, (3) the mean ± 1 standard deviation of the predicted C contents and (4) the median ± 1 standard deviation of the predicted C contents.

The incorporation of the uncertainty in model evaluation reflects the fact that the predicted C content represents only a 2D surficial cross-section of the core, whereas the C analysis was performed on a small subsample of the larger homogenized 3D core half. As such, despite sample homogenization, the subsamples analysed for C content on the bulk samples do not completely correspond with the surface C content of the core half predicted from hyperspectral imaging.
